# Effects of Dietary Protein Levels, Net Energy Levels, and Essential Amino Acid-to-True Protein Ratios on Broiler Performance

**DOI:** 10.3390/ani14213065

**Published:** 2024-10-24

**Authors:** Sosthene Musigwa, Pierre Cozannet, Collins A. Asiamah, Shu-Biao Wu

**Affiliations:** 1 Animal Science, School of Environmental and Rural Science, University of New England, Armidale, NSW 2351, Australia; casiamah@myune.edu.au; 2 Center of Expertise and Research in Nutrition (CERN), Adisseo France SAS, 92160 Antony, France; pierre.cozannet@adisseo.com

**Keywords:** essential-to-true protein ratio, crude protein, net energy, reduced-protein diet, true protein

## Abstract

Supplementing essential amino acids (EAAs) without considering non-EAA (NEAA) and energy contents in reduced-crude protein (CP) diets may alter EAA-to-true protein (E:T) and energy-to-protein ratios, potentially compromising growth. This study aimed to evaluate the effects of CP, E:T, and net energy (NE) on broiler performance. The treatments were as follows: T1—reduced CP (16%, RP), low NE (9.9 MJ/kg, LNE), low E:T (0.56, LE:T); T2—RP, LNE, high E:T (0.60, HE:T), with imbalanced EAA (excess Met and deficient Thr); T3—RP, high NE (10.4 MJ/kg, HNE), LE:T; T4—RP, HNE, HE:T; T5—normal CP (18%, NP), LNE, LE:T; T6—NP, LNE, HE:T; T7—NP, HNE, LE:T; and T8—NP, HNE, HE:T. The study employed as-hatched Cobb 500 broilers in two experiments. Exp.1 studied the broiler performance from d19 to 35, with eight replicates per treatment and 16 birds per replicate (*n* = 1024). Birds were randomly assigned to different treatments, and at the end of their lives, all were dissected to determine their sex and account for any gender-related effects. Exp.2 measured NE values in respiration chambers from d25 to 28, with six replicates per treatment, and two birds (a male and a female) per replicate (*n* = 96). The bird gender was determined through high-resolution melting curve analysis of feather DNA. The measured NE values were used to calculate NE intake (NEi) in Exp.1. The results showed that T4 improved (*p* < 0.001) weight gain (WG), feed conversion ratio (FCR), and NEi relative to T1, T2, and T3, and protein efficiency (WG/CP intake) relative to all treatments. The live performance (feed intake, WG, FCR) and NEi of birds fed T4 reached a level equal to those of birds fed NP diets (T5 to T8). These results suggest that a dietary E:T ratio of 0.60 is necessary to maximize nutrient utilization and to restore growth rate in broilers fed RP diets.

## Simple Summary

Dietary energy and protein are the most critical nutrients for broilers. Excessive levels of these nutrients can lead to negative economic and environmental consequences, while insufficient amounts hinder growth and feed efficiency. Therefore, accurately estimating these nutrients is vital, particularly in reduced-protein diets. In such diets, essential amino acids are commonly supplemented without considering non-essential amino acids, which can alter the essential-to-total amino acid (true protein) ratios and negatively impact bird performance. There is a notable inconsistency in the limited studies available regarding the optimal essential-to-total amino acid ratio in poultry diets. Thus, this study was designed to investigate the effects of different essential-to-total amino acid ratios and energy levels on the performance in broilers fed reduced-protein diets from day 19 to 35. The objective was to determine the optimal essential-to-total amino acid ratio and net energy value in reduced-protein diets. The results suggest that the reduced-protein diets containing 60% essential and 40% non-essential amino acids promotes maximum nutrient utilization and supports similar growth compared to broilers fed conventional diets.

## 1. Introduction

Dietary energy and protein constitute the most predominant nutrients, representing approximately 90% of the overall cost of broiler diets [1]. Excessive concentrations of energy and protein cause adverse economic and environmental effects, while insufficient concentrations detrimentally affect growth performance and feed efficiency [2]. Therefore, the accurate estimation of these nutritional elements is crucial, especially when formulating reduced-crude protein (RP) diets. This precision can help to enhance nutrient utilization efficiency, improve live performance, and maximize economic returns in broilers [3].

The apparent metabolizable energy (ME) system is conventionally used to determine the dietary energy values of poultry feedstuffs. However, this system tends to undervalue fats and overestimate proteins by neglecting heat increment (HI) as an energy loss. Conversely, the net energy (NE) system accounts for HI loss during metabolism, providing a more accurate approach to estimate feed energy [4].

Understanding dietary protein intake is crucial for maintaining proper nitrogen (N) levels. Crude protein (CP) is often used to estimate protein content in feed, but it tends to overestimate the true protein (TP) content [5]. This is because CP uses a standard N-to-protein conversion factor of 6.25, assuming all proteins have 16% N, which ignores variations in amino acid (AA) composition and non-protein N compounds. Natural ingredients contain non-AA N sources like nucleic acids, urea, and ammonia [6], which are only partly useful for monogastric animals. Therefore, focusing on AA N gives a more accurate measure of dietary protein. TP content, which includes essential (EAA) and non-EAA (NEAA), is calculated using a specific conversion factor, K_A_ [7]. The ratio of EAA to TP (E:T) simplifies the complex relationship between these AAs [6,8].

Reducing dietary CP levels and supplementing with EAA without considering NEAA can disrupt the E/T ratio, negatively impacting growth, as this ratio in poultry tissues remain constant [9]. Bedford et al. [10] observed that the EAA:NEAA ratio influences growth by affecting feed intake (FI). Similarly, Peres et al. [11] found that the FI in fish increased as E/T ratios decreased to maintain a constant EAA intake. Achieving the optimal E/T ratio maximizes protein utilization efficiency. At low ratios, N utilization decreases due to a surplus of NEAAs that are degraded and excreted. Conversely, when the ratio exceeds the optimal level, N utilization also declines due to NEAA deficiency [6]. Thus, maintaining an optimal E/T balance in poultry diets is crucial for maximizing protein utilization and growth performance.

Investigations into the optimal balance of dietary AA for optimum growth have revealed a wide spectrum of E:T values in kittens. These values range from 0.3 to 0.9 E:T in diets containing 10% to 55% CP, provided that the excess AA leading to growth depression is mitigated [6]. Young rats were also found to be insensitive to changes in dietary E:T, suggesting that optimal performance can be achieved in diets containing E:T ratios ranging from 0.50 to 0.80 [12]. However, chickens are more sensitive to dietary E:T ratio changes than rats and kittens [12]. Therefore, efforts have been made to determine the optimal E:T ratio for poultry, yielding a variety of results. For instance, the optimal E:T ratios found for broilers in different studies were 0.48 [13], 0.50 [14,15], and 0.55 [12]. Bedford et al. [10] found the optimal ratio to be 0.60 for maximum growth in turkeys. Given these noticeable inconsistencies among a limited number of studies, a more in-depth investigation is warranted. 

This study was designed to explore the effects of E:T and NE on the performance and carcass characteristics of broilers from d19 to 35. This study aimed to identify the optimum E:T ratio and NE value in RP diets for broilers. We hypothesized that an optimum E/T ratio in RP diets containing low energy densities would enhance bird performance.

## 2. Materials and Methods

The animal ethics committee of the University of New England approved the procedures conducted in this study (authority no: ARA22-032).

### 2.1. Experimental Design and Treatment Diets

Eight dietary treatments were formulated in this study. The first four treatments (T1 to T4) had a reduced CP content (16%), while the last four (T5 to T8) had a normal CP content (18%). Each group (RP or NP diets) was further divided into two subgroups based on two levels of NE (9.9 or 10.4 MJ/kg) and two levels of E:T ratio (0.56 or 0.60) as shown in Table 1. This study was initially designed as a 2 × 2 × 2 factorial arrangement of treatments to assess the effects of CP, E:T, and NE on performance. However, an error during feed mixing resulted in an imbalance of Met and Thr, with excess Met and deficient Thr in the analyzed values in T2. However, this treatment was retained to demonstrate how the imbalance in Met and Thr affects performance and energy utilization. Consequently, the study was analyzed using one-way ANOVA due to this imbalance. The study was undertaken in two experiments using same diets. Exp.1 measured bird performance in floor pens, and Exp.2 studied energy partitioning in closed respiration chambers.

All diets were formulated to meet or exceed the nutrient specifications outlined in the Cobb 500 guidelines [16], except for CP and NE (and additionally, Thr and Met in T2) in the experimental diets. The diets were supplemented with exogenous feed enzymes, including carbohydrases and phytases, factoring in their matrix values to ensure alignment with standard commercial broiler diets. The compositions and calculated nutrient levels of the experimental wheat/barley/soybean meal-based diets are presented in Table 2 and Table 3. The analyzed dietary nutrients are shown in Table 4.

A lysine content of 0.995% was determined by averaging Cobb finisher 1 and finisher 2 values [16]. The other AA requirements were calculated based on the ideal protein concept from the Texas A&M ratios [17]. This procedure involved keeping the ratio of each EAA to lysine consistent. All diets were supplemented with crystalline AA to ensure that bird requirements were met for all EAA, except T2. NE was reduced by decreasing the oil supplementation content. The E:T ratio was utilized as a tool to balance NEAA. Specifically, a ratio of 0.56 was indicative of high NEAA contents in diets, while a ratio of 0.60 signified low NEAA concentrations. RP and normal CP (NP) diets were isonitrogenous, with 16 and 18% CP, respectively (total N × 6.25). This means that any changes in the E:T value occurred at a constant concentration of CP.

Crystalline NEAA supplements were employed to reduce the E:T ratio. NEAAs supplemented in diets include glycine, alanine, aspartate (aspartic acid), glutamate (glutamic acid), glutamine, and proline. The true protein (TP = EAA + NEAA) contribution of each ingredient during feed formulation (other than purified AA) was estimated using a specific N-to-protein conversion factor, also known as *K*_A_, which was sourced from the literature [18].

A *K*_A_ value of 6.25 was used for all purified AA used in the feed formulation [9]. The CP values of commercial AA were estimated based on Tillman [19]. Thus, the TP value was estimated according to Alhotan et al. [8] as follows:

Ingredient CP contribution (%) to total CP = ingredient CP content (%) × amount of ingredient used (%).

Ingredient total N (%) = CP content %/6.25.

Ingredient TP contribution (%) to feed TP = ingredient total N × ingredient *K*_A_.

Glycine equivalent (Gly_equiv_) was maintained above 1% across treatments and was calculated as follows [20]:

Gly_equiv_ (%) = Gly (%) + [0.7143 × Ser (%)],

where 0.7143 is the ratio of the molar weight between Gly and Ser. Glycine was incorporated into the diets as the NEAA source.

**Table 3:** Calculated nutrient composition (in % unless otherwise indicated).

Nutrient, %	Starter	Grower	T1	T2	T3	T4	T5	T6	T7	T8
AMEn, MJ/kg	12.45	12.66	12.46	12.43	13.02	12.99	12.45	12.39	13.01	12.99
NE, MJ/kg	9.9	10.0	9.9	9.9	10.4	10.4	9.9	9.9	10.4	10.4
CP (N × 6.25)	23	21	16	16	16	16	18	18	18	18
TP (N × K_A_)	19.00	18.27	13.77	13.21	13.78	13.15	15.05	14.10	15.09	14.07
EAA	10.49	9.71	7.72	7.94	7.72	7.94	8.42	8.44	8.45	8.44
Crude fat	6.49	5.23	3.37	4.16	4.92	5.59	4.63	6.34	6.38	6.84
Crude Fiber	4.85	4.74	4.80	4.94	4.27	4.30	4.44	5.86	4.30	4.48
d Gly _equiv_^1^	1.383	1.307	1.050	1.050	1.050	1.050	1.028	1.019	1.041	1.029
d Arg	1.280	1.180	1.075	1.075	1.075	1.075	1.075	1.075	1.075	1.075
d Lys	1.220	1.120	0.995	0.995	0.995	0.995	0.995	0.995	0.995	0.995
d Met ^2^	0.569	0.509	0.418	0.619	0.418	0.418	0.418	0.418	0.418	0.418
d M+C	0.311	0.297	0.328	0.709	0.328	0.328	0.328	0.328	0.328	0.328
d Trp	0.880	0.806	0.746	0.746	0.746	0.746	0.746	0.746	0.746	0.746
d Leu	0.278	0.259	0.169	0.176	0.169	0.176	0.217	0.220	0.218	0.221
d Ile	0.895	0.847	0.597	0.597	0.597	0.597	0.677	0.673	0.686	0.685
d Tyr	1.372	1.290	1.085	1.085	1.085	1.085	1.085	1.085	1.085	1.085
d Asn	0.820	0.766	0.678	0.678	0.678	0.678	0.678	0.678	0.678	0.678
d Thr ^2^	0.648	0.621	0.314	0.439	0.313	0.379	0.479	0.464	0.490	0.486
d Val	0.767	0.708	0.285	0.382	0.285	0.381	0.527	0.519	0.541	0.538
d Gly	0.817	0.750	0.697	0.697	0.697	0.697	0.697	0.710	0.697	0.697
d Ser	0.939	0.860	0.796	0.796	0.796	0.796	0.796	0.796	0.796	0.796
d Pro	0.758	0.705	0.751	0.689	0.753	0.690	0.567	0.577	0.570	0.565
d Ala	0.876	0.843	0.418	0.505	0.416	0.504	0.645	0.619	0.659	0.650
d Asp	1.214	1.203	1.033	0.902	1.027	0.907	1.037	1.008	1.040	1.028
d Glu	0.820	0.761	0.628	0.529	0.628	0.517	0.643	0.596	0.643	0.632
d Phe + Tyr	1.126	1.080	0.631	0.563	0.628	0.566	0.762	0.742	0.796	0.797
d Gln	2.370	2.517	1.820	1.568	1.796	1.610	1.878	1.881	1.921	1.936
Starch	1.537	1.461	0.709	0.874	0.706	0.872	1.127	1.090	1.150	1.142
Calcium	0.880	0.800	0.760	0.760	0.760	0.760	0.760	0.760	0.760	0.760
P available	0.440	0.400	0.380	0.380	0.380	0.380	0.380	0.380	0.380	0.380
Sodium	0.160	0.160	0.220	0.220	0.220	0.220	0.220	0.220	0.220	0.220
Potassium	0.950	0.878	0.950	0.950	0.950	0.950	0.950	0.950	0.950	0.950
Chloride	0.300	0.203	0.300	0.300	0.300	0.300	0.300	0.300	0.300	0.300
Choline, mg/kg	1700	1500	1500	1500	1500	1500	1500	1500	1500	1500
Linoleic 18:2	2.075	1.790	1.271	1.478	1.654	1.831	1.597	1.929	2.037	2.100
DEB (Na+K-Cl) ^3^	228	237	254	254	254	254	254	254	254	254
E:T	0.55	0.53	0.56	0.60	0.56	0.60	0.56	0.60	0.56	0.60

Abbreviations: AMEn, apparent metabolizable energy corrected of nitrogen; NE, net energy; CP, crude protein; TP, true protein; K_A_, ingredient-specific N-to-protein conversion factor; EAA, Essential amino acids; E:T, EAA-to-TP ratio. ^1^ Gly_equiv_, Glycine equivalent (%) = Gly (%) + [0.7143 × Ser (%g)], where 0.7143 is the ratio of the molar weight between Gly and Ser [20]. ^2^ Analyzed concentrations of Met and Thr in T2. ^3^ DEB, dietary electrolyte balance (mEq/kg) = Na/0.0023 + K/0.00391 − Cl/0.00355).

**Table 4:** Analyzed concentrations of AA (% as is), CP (% as is), and energy1 in experimental diets.

Measured Nutrients, %	T1	T2	T3	T4	T5	T6	T7	T8
Lysine	0.999	1.041	1.028	0.947	1.041	1.078	1.138	1.152
Methionine	0.395	0.619	0.355	0.371	0.414	0.354	0.395	0.356
Threonine	0.680	0.439	0.683	0.690	0.749	0.763	0.776	0.755
Arginine	1.006	1.057	1.022	1.057	1.047	1.004	1.085	1.044
Phenylalanine	0.634	0.658	0.651	0.641	0.722	0.704	0.768	0.808
Valine	0.830	0.886	0.831	0.860	0.880	0.884	0.909	0.925
Isoleucine	0.694	0.763	0.689	0.692	0.688	0.698	0.715	0.757
Leucine	1.173	1.215	1.159	1.168	1.175	1.182	1.229	1.267
Histidine	0.263	0.326	0.272	0.321	0.401	0.403	0.419	0.420
Serine	0.473	0.589	0.469	0.571	0.719	0.728	0.748	0.766
Glycine	0.813	0.780	0.794	0.740	0.669	0.688	0.678	0.681
Aspartic acid	0.882	0.991	0.900	0.940	1.254	1.203	1.335	1.354
Glutamic acid	3.055	2.732	3.017	2.660	3.190	3.196	3.361	3.605
Alanine	0.673	0.605	0.668	0.554	0.679	0.640	0.699	0.705
Proline	1.045	0.940	1.037	0.929	1.102	1.068	1.132	1.231
Tyrosine	0.242	0.248	0.195	0.293	0.384	0.320	0.403	0.338
CP	15.58	15.41	15.58	15.07	17.45	16.66	17.07	17.74
AME ^1^, MJ/kg	13.89	13.98	14.40	14.43	13.88	13.61	14.33	14.20
AMEn ^1^, MJ/kg	13.27	13.42	13.78	13.78	13.08	12.91	13.60	13.45
NE ^1^, MJ/kg	10.23	10.55	10.70	11.12	10.41	10.29	10.67	10.63

Abbreviations: AA, amino acids; CP, crude protein; AME, apparent metabolizable energy; AMEn, AME corrected of nitrogen; NE, net energy. ^1^ AME, AMEn and NE (MJ/kg DM) were analyzed in closed respiration chambers from d25 to 28.

### 2.2. Birds and Housing Management

The husbandry practices (lighting program and temperature) were based on Cobb 500 management guidelines [21]. This study employed as-hatched d-old Cobb 500 broiler chicks obtained from a commercial hatchery (Baiada Poultry Pty Ltd., Tamworth, NSW, Australia) in two experiments. These include a floor pen performance trial (Exp.1) and a calorimetric trial (Exp.2). For both experiments, birds were offered feeds and water ad libitum throughout the study. The birds were fed identical dietary compositions in three phases, namely, a common starter diet (d0 to 8), a common grower diet (d9 to 18), and finisher treatment diets from d19.

In Exp.1 (d19 to 35), 1024 birds were housed in 64 pens, and each treatment was replicated in 8 pens of 16 birds per pen to study the performance. Bird and feed weights were recorded on d19, 28, and 35. On d28, 4 birds per pen (2 males and 2 females) were sampled and euthanized via electrical stunning followed by cervical dislocation to collect ileal digesta for N digestibility evaluation. Ileum digesta contents from 4 birds were collected by gentle digital pressure, pooled into one pot per pen, and stored at −20 °C prior to freeze drying. On d35, an additional 4 birds per pen (2 males and 2 females) were sampled and euthanized via electrical stunning followed by cervical dislocation to weigh carcass parts. At the end of their lives, all birds were dissected to determine their sex to account for any gender-related effects.

In Exp.2 (d21 to 28), a total of 96 birds were subjected to the calorimetric trial, which was conducted three times using 16 closed respiration chambers. Each chamber housed 2 birds (one male and one female). The sex of all birds used in the calorimetric chambers was determined through a high-resolution melting curve analysis of feather DNA [22]. From d0 to 21, the birds were raised in floor pens within a climate-controlled room. Subsequently, they were acclimatized to the calorimetry chambers from d21 to 25. The calorimetric measurements took place from d25 to 28, during which total excreta was collected, and the weights of birds, feed, and O_2_ cylinders were recorded. Respiratory gas exchange was measured daily and per chamber for NE measurements [23]. Total fresh excreta were daily collected per chamber in a labeled plastic box with a lid and stored in fridge (4 °C) following collections. On day 28, the excreta box from each chamber was weighed and thoroughly mixed. A subsample was collected in a pot and then stored at −20 °C before being freeze-dried to a constant weight.

### 2.3. Laboratory Analysis and Calculations

Diet and digesta samples were subjected to dry matter (DM) analysis through oven drying at 105 °C until a consistent weight was achieved. AA concentrations in the diets were determined employing the Waters AccQTag AA analysis methodology adapted for ultra-performance liquid chromatography (UPLC) with an ACQUITY UPLC system and a UV detector (Waters Corporation, Milford, MA, USA) [24,25]. The TiO_2_ concentration in diets and digesta samples was assessed following the protocol outlined by Short et al. [26]. The apparent ileal digestibility coefficient (dc) was calculated as follows:

dc = 1− [TiO_2_diet (%)/TiO_2_ digesta (%)] × [AA digesta (%)/AA diet (%)].

Freeze-dried and ground excreta and feed samples in Exp.2 were analyzed for gross energy (GE) utilizing a Parr 6400 automatic isoperibol calorimeter (Moline, IL, USA). Additionally, N content was determined using a LECO^®^ FP-2000 automatic N analyzer (Leco Corporation, St. Joseph, MI, USA). The analysis of KOH samples for CO_2_ recovery was conducted using the BaCl_2_ precipitation method as outlined by Annison et al. [27]. The volumes, measured in liters, of O_2_ consumed and CO_2_ produced were employed in the calculation of heat production (HP, kcal) based on the modified Brouwer [28] equation.

HP = 1.200 × CO_2_ + 3.866 × O_2_

Feed AME (kcal/kg DM) was calculated using the following equation:

AME = [(feed GE × FI) − (excreta GE × total excreta output)]/FI

Retained energy (RE) was obtained by subtracting heat production (HP) from AME intake (AMEi), and NE was calculated as RE plus fasting HP. The fasting HP value used was 450 kJ per metabolic body weight (BW^0.70^). Feed NE concentration was calculated by dividing NEi by FI [29]. The dietary AME and NE values from Exp.2 were used to determine the corresponding AMEi and NEi values in Exp.1.

### 2.4. Statistical Analysis

Data were statistically analyzed in a random design using a one-way ANOVA on the JMP Pro 18 (SAS Institute Inc., JMP Software, Cary, NC, USA, 2019) standard least squares (LS) personality. The percentage of male birds functioned as a covariate in Exp.1, while in Exp.2, the run variable served as a covariate. All non-normally distributed data were transformed using the fitted distribution function of JMP prior to analysis. The LS means Tukey honestly significant difference (HSD) option was applied to determine significantly different means. The experimental unit was the pen mean for Exp.1 and the chamber mean for Exp.2, considering a 5% level of probability to be significant. The correlations between the analyzed values of dietary CP, TP, EAA, and NE) and the measured parameters were estimated using a JMP multivariate correlation analysis.

## 3. Results

### 3.1. Growth Performance and Energy Utilization from d19 to 28

The effects of dietary treatments on bird performance (d19 to 28), nutrient utilization and fat pad weight (d28) are presented in Table 5. In the NP diet group (T5 to T8), birds fed T6 (with LNE) had a decreased (*p* < 0.001) N dc and increased (*p* < 0.001) FCR compared to T8 (HNE), likely due to a reduction in dietary NE density. In addition, the T5-fed birds had a lower (*p* < 0.001) protein efficiency (WG/CP intake) than other counterparts fed NP diets, and the birds fed T5 and T6 had similar (*p* > 0.05) FCR. Similarly, there was no significant differences (*p* > 0.05) in FCR and WG/CP intake between birds fed T7 and T8, but those fed T7 had a lower (*p* < 0.001) N dc than those on T8. All other measured variables (FI, WG, AMEi, NEi, AMEi/WG, NEi/WG, and abdominal fat pad) remained similar (*p* > 0.05) irrespective of the NE and E:T contents of the NP diets.

In birds fed RP diets, there was no difference (*p* > 0.05) between T1 and T3 (low E:T) on the measured responses (irrespective of the NE density), except for protein utilization efficiency, where increasing NE in T3 led to the improved (*p* < 0.001) protein efficiency compared to low NE in T1. However, all the measured variables except (*p* > 0.05) for the NEi/WG and fat pad values were negatively affected (*p* < 0.001) in T3 relative to T4 due to the decreased E:T ratio from 0.60 to 0.56. Birds fed T4 had a better (*p* > 0.001) WG, FCR, energy intake (both for the AME and NE system), AMEi/WG, WG/CP intake, and lower N dc compared to the other RP group. In addition, the T4-fed birds had a similar (*p* > 0.05) WG, FI, and N dc, but a higher (*p* < 0.001) WG/CP intake relative to the NP-fed birds. Except for fat pad content (*p* > 0.05), all measured variables were significantly negatively affected (*p* < 0.001) in T2 compared to T4, likely due to an imbalance between Met and Thr in T2. Although there were no significant differences in WG, FCR, fat pad, and N dc between T2 and T1, the remaining variables were negatively affected in birds fed T2 compared to those fed T1, also likely due to the imbalance between Met and Thr in T2.

### 3.2. Growth Performance and Energy Utilization from d19 to 35

The effects of CP, NE, and E:T on bird performance, energy utilization, and carcass quality from d19 to 35 are shown in Table 6. All the measured variables did not differ (*p* > 0.05) among birds fed NP diets, regardless of the varying levels of NE or E:T. For birds fed RP diets, the NE densities in the 0.56 E:T RP diets (T1 vs. T3) showed no difference (*p* > 0.05) in any of the measured responses. In the HNE RP diets, elevating E:T from 0.56 (T3) to 0.60 (T4) improved (*p* < 0.001) the WG, FCR, NEi, AMEi/WG, and WG/CP intake in birds fed T4 relative to T3 and to all the other RP diets. However, FI, AMEi, NEi/WG, breast yield, and fat pad remained unaffected (*p* > 0.05) by E:T ratios between birds fed T4 and T3. Imbalanced Met and Thr in T2 severely affected FI, energy cost effectiveness (AMEi/WG and NEi/WG) and WG/CP intake relative to T1. This imbalance also negatively affected all the measurements except for fat pad in birds fed T2 relative to those fed T4. The overall mortality during the experimental period was less than 2%, and there was no dietary treatment-related mortality (*p* > 0.05).

### 3.3. Correlations Between the Experimental and Measured Variables (d19 to 35)

Correlations between the experimental and measured variables from d19 to 35 are presented in Table 7. WG was positively correlated (*p* < 0.001) with energy intake (*r* = 0.876 for AMEi, and *r* = 0.864 for NEi), dietary CP % (*p* < 0.001, *r* = 0.591), dietary TP % (*p* < 0.001, *r* = 0.583), and dietary EAA % (*p* < 0.001, *r* = 0.444). However, WG was not correlated (*p* > 0.05) with dietary AME or NE content. In addition, the dietary EAA was negatively correlated (*p* < 0.001) with FCR, (*r* = −0.492) and fat pad (*r* = −0.626), and positively correlated with breast yield (*p* < 0.001, *r* = 0.689). NEi was weakly correlated with dietary NE (*p* < 0.05, *r* = 0.312) but was not correlated with dietary AME (*p* > 0.05).

## 4. Discussion

Maintaining an optimal balance of AA and energy in poultry diets is essential for maximizing nutrient utilization and supporting growth. The present study aimed to investigate the impact of dietary E:T and NE levels on the performance of broilers fed RP diets. The average WG from d19 to 35 in birds fed NP diets (111.64 g/b/d for T5 to T8) and the HNE RP diet with a 0.60 E:T ratio (T4, 106.76 g/b/d) exceeded the Cobb 500 2022 breeders’ standard (98.50 g/b/d), suggesting that the adequacy of breed nutrient specifications was met. 

### 4.1. Influence of E:T Ratios in NP Diets on the Measured Variables 

Data from the present study demonstrated that E:T ratios did not affect the measured responses (FI, WG, FCR, NEi, and NEi/WG from d19 to 35, and breast, and fat pad at d35 in broilers fed NP diets (18% CP, T5–T8). Similar findings have previously shown that though the optimum E:T ratio is required for maximum protein utilization for growth, changes in E:T levels are unlikely to influence performance in normal or high CP diets, where the levels of total AA N are above the minimum requirements [15]. In contrast, E:T values below or above the optimum in RP diets, where the excess of total AA N is minimized, may lead to a depressed performance due to an imbalance in the EAA:NEAA ratio [6,14,30]. This hypothesis is consistent with previous research on broilers showing that for each increase in the EAA:NEAA ratio above the optimum, the rate of EAA intake decreased in broilers fed RP diets (14% and 18% CP). However, with 22% CP diets, the EAA intake consistently increased with an increasing EAA:NEAA ratio [12]. The finding that NE levels did not influence the live performance in birds fed NP diets partly agrees with the work of Infante-Rodríguez et al. [31] who observed that varying dietary energy from 3040 to 3160 kcal/kg AME did not influence the weight of broilers fed 18.7% CP diets, though their FI was reduced.

### 4.2. Effect of E:T Levels in RP Diets on the Measured Responses

When the E:T was increased to 0.60 in T4 (HNE RP diet), WG, FCR, and NEi were maximized relative to T3 (HNE RP diet at 0.56 E:T). In addition, the live performance (FI, WG, FCR) and energy utilization (AMEi, and NEi) of birds fed T4 reached levels equal to those fed the NP diets. These findings support the Waldroup et al. [32] hypothesis that bird growth potential is driven by FI, which can be enhanced by limiting excessive AA in diets, ultimately leading to an improved performance. This notion was confirmed by Leeson et al. [33], who observed that a slower growth of pullets fed 13% CP was associated with a reduced FI relative to those fed the 18% grower diet. Additionally, Almquist [34] stated that when the protein in the diet is precisely balanced and present in adequate amounts, the rate of tissue synthesis and the effectiveness of utilizing the diet for growth will approach a maximum determined by the animal genetic potential. This could explain the adverse effects on live performance commonly reported in the literature [35,36]. Reducing dietary CP content and simply adding EAA to meet requirements, without considering NEAA contents, may lead to an E:T imbalance, which could be one of the factors contributing to poor performance [12,37]. Recently, Camiré et al. [38] recommended using the EAA N-to-total AA N ratio to illustrate the complex relationship between EAA and NEAA and to indicate an adequate supply of these AAs. 

The birds fed the T4 diet (RP-HNE-HE:T) demonstrated better protein utilization efficiency (WG/CP intake) compared to those fed other RP and NP diets throughout the entire experimental period. This indicates that less N was excreted, as suggested by Bregendahl [39]. These findings confirm the importance of defining the optimal dietary EAA/NEAA ratio to maximize protein utilization efficiency and overall performance, as previously noted [6,11]. Green et al. [30] emphasized the importance of maintaining an optimal dietary EAA:NEAA ratio for achieving optimal growth and efficient protein utilization. Corzo et al. [40] also highlighted that protein utilization is most efficient when all AA are at or slightly below, but not above, their required levels for protein synthesis. It is worth mentioning that although the birds fed T4 exhibited the highest protein utilization efficiency among all treatments, their FCR was still poorer compared to those fed T7 (NP-HNE-LE:T) and T8 (NP-HNE-HE:T) diets. This aligns with the observation by Roosendaal et al. [41], who stated that the efficiency of nutrient conversion, based on the product objective, should take precedence over FCR as a response criterion. 

Additionally, the E:T value of 0.60 shown herein to maximize the live performance in broilers fed RP diets is consistent with the ratio corresponding to the maximum FI and growth in turkeys [10] and to the maximum N retention in growing pigs (0.61 E:T) when the total N concentration was kept constant [42], to the maximum protein and energy utilization (EAA:NEAA ratio of 60:40 or 0.60 E:T) in the European sea bass fish [11], and to the maximal FI, WG, feed efficiency ratio, and N retention (57:43 EAA:NEAA) in the rainbow trout fish [30]. In contrast, Heger [6] reported that E:T ratios ranging from 0.55 to 0.60 are required for optimum growth across species, including chickens, turkeys, rats, and pigs. In this study, compared with the 0.60 E:T ratio, the 0.56 E:T ratio was associated with depressed bird performance. These discrepancies are probably due to differences in EAA classification and methodological approaches used for E:T estimations [6,14], or other dietary factors, such as an imbalance within EAA contents in diets.

Furthermore, a 0.60 E:T ratio in the HNE RP diet (T4) enhanced NEi relative to a 0.56 HNE RP diet (T3), but AMEi remained unaffected. This finding suggests that energy intake is better predicted by the NE system than the AME system, as demonstrated by a correlation between feed NE and NEi, and the absence of correlation between AME and NEi. The fact that a 0.60 E:T ratio improved NEi confirms the findings from Classen [43], who suggested that bird responses to dietary energy can be affected by AA balance, and Nieto et al. [44], who found that an improved dietary protein quality affects the efficiency of energy utilization. The improved NEi herein might have contributed to the observed improvement in WG in birds fed T4 compared to those fed T3. This is further demonstrated by a strong correlation between WG and NEi. In support of this, Close et al. [45] stated that the benefits resulting from an increase in protein intake are apparent only when there is sufficient availability of dietary energy. Additionally, the absence of CP effect on the live performance between birds fed NP diets and RP-HNE diet with 0.60 E:T (T4) has substantiated the hypothesis of Bedford et al. [12]. According to this, broilers have a specific need for how their total dietary protein content is divided into EAA and NEAA, rather than the quantity of protein per se they receive.

While T4 (16% CP) restored the live performance to that of conventional diets (18% CP), NEi/WG, breast yield, and fat pad remained unimproved. A reduction in dietary protein led to an increase in abdominal fat regardless of energy density, highlighting that dietary AA content, not NE concentration, influences fat pad content. This was further demonstrated by the negative correlations between abdominal fat pad and dietary EAA, TP, and CP, but not with dietary NE. This finding is in line with that of Waldroup et al. [46], who reported that dietary energy content did not influence abdominal fat pad weight. However, it was previously demonstrated that protein retention depends on the rate of energy supply. Once animals reach their maximum potential for protein deposition, the excess energy is then utilized for lipid synthesis. This implies that there is still an indirect energy–protein relationship, wherein birds fed RP diets have more excess energy after accounting for the energy cost of protein deposition than those fed NP diets. The finding that dietary CP decreases with increasing abdominal fat pad is consistent with the findings of previous studies [36,47]. However, this was not consistent with Kamran et al. [48], who found that abdominal fat pad weight levels remained similar among broilers fed diets containing 17% to 23% CP from d1 to 35. Further research is needed to understand the energy–protein relationship on body fat in RP diets.

### 4.3. Effect of NE Levels in NP and RP Diets on the Measured Responses

Data from the present study demonstrated that NE densities did not affect the measured responses (FI, WG, FCR, NEi, NEi/WG, breast and fat pad from d19 to 35) in broilers fed NP diets. The finding that NE levels did not influence the live performance in birds fed NP diets partly agrees with the work of Infante-Rodríguez et al. [31], who observed that varying dietary energy from 3040 to 3160 kcal/kg AME did not influence the weight of broilers fed 18.7% CP diets, though their FI was reduced.

Similarly to NP diets, the NE contents in the 0.56 E:T ratio RP diets (T1 vs. T3) did not influence any of the measured variables. This result is partly in accordance with the findings of Classen [43], who observed that dietary energy levels did not affect response criteria and concluded that energy levels had no effect on diets containing high or moderate AA. It has previously been shown that chickens adjust their FI in an attempt to maintain a constant level of energy intake rather than AA intake [49,50]. This hypothesis held true solely for the NP diets in this study, where dietary NE levels did not influence the NEi. In the RP diets, however, NEi varied with dietary NE contents, reaching its maximum in the RP-HNE diet at a 0.60 E:T ratio. This is partly in accordance with the findings of Classen [43], who observed that broilers are unable to adjust their FI to match energy requirements, and Parr et al. [51], who found that the control of FI is highly influenced by dietary AA to maintain EAA intake.

The difference in FI observed between the RP-LNE diet with a 0.60 E:T ratio (T2) and the HNE RP diet with a 0.60 E:T ratio (T4) was most likely due to the imbalance between Met and Thr in T2, rather than the difference in NE content. The resulting depressed FI observed in birds fed this treatment led to impairments in NEi, WG, FCR, and NEi/WG compared to those fed T4. Similarly to this, Waldroup et al. [52] showed that feeding animals with diets containing imbalanced AA results in a depressed FI. Lysine imbalances (both a deficiency and excess) affect the growth rate and the energy utilization efficiency in broilers and turkeys [53]. Therefore, the impact of NE levels in 0.60 E:T ratio RP diets observed in this study is inconclusive due to the imbalance within EAA in T2. Thus, further investigation is needed to understand the NE effect in 0.60 E:T ratio RP diets.

## 5. Conclusions and Implications

In conclusion, the data from the current study confirm that achieving a balance between dietary EAA and NEAA is feasible. The recommended optimum E:T ratio to maximize protein utilization in RP diets for broiler growth is approximately 0.60, equivalent to 60% EAA and 40% NEAA of total AA. Increasing the E/T ratio up to 0.60 in RP feed formulations can maximize protein utilization. This can lead to more efficient growth in broilers and overall growth performance. In addition, a proper E/T balance in feeds can reduce nitrogen excretion into the environment. Thus, a 0.60 E/T ratio could contribute to more sustainable poultry farming practices. Further studies are necessary to examine the precise energy-to-protein balance required for reducing body fat content in broilers fed RP diets.

## Figures and Tables

**Table 1:** Description of dietary treatments.

Treatment	Treatment Code	Dietary Treatment Description
1	RP-LNE-LE:T	Reduced CP (16%) with low NE (9.9 MJ/kg) and low E:T (0.56)
2	RP-LNE-HE:T	Reduced CP with low NE, high E:T ratio (0.60) and imbalanced Met and Thr ratio
3	RP-HNE-LE:T	Reduced CP with high NE (10.4 MJ/kg) and low E:T ratio
4	RP-HNE-HE:T	Reduced CP with high NE and high E:T ratio
5	NP-LNE-LE:T	Normal CP (18%) with low NE and low E:T ratio
6	NP-LNE-HE:T	Normal CP with low NE and high E:T ratio
7	NP-HNE-LE:T	Normal CP with high NE and low E:T ratio
8	NP-HNE-HE:T	Normal CP with high NE and high E:T ratio

Abbreviations: CP, crude protein; E:T, essential amino acid-to-true protein ratio; NE, net energy.

**Table 2:** Feed ingredients used.

Ingredients, %	Starter	Grower	T1	T2	T3	T4	T5	T6	T7	T8
Wheat	17.3	27.9	28.6	20.0	27.8	22.3	20.0	22.8	20.0	20.0
Barley	20.0	20.0	25.0	25.0	25.0	25.0	25.0	25.0	25.0	25.0
Soybean meal	27.2	26.2	3.0	7.9	2.8	7.8	13.7	12.2	15.7	15.8
Wheat Pollard	9.9	5.0	8.1	11.0	8.0	11.4	9.4	16.5	9.1	17.0
Sorghum	1.0	1.0	10.0	13.0	10.0	10.0	8.3	2.0	6.6	8.3
Corn	10.0	10.0	10.0	10.0	10.0	10.0	10.0	2.0	10.0	2.0
Canola ml solvent	4.0		0.5	0.5	1.0	0.5	4.2	6.0	2.3	0.5
Canola oil	4.80	3.56	1.54	2.25	3.09	3.74	2.73	4.81	4.57	5.25
Rice hulls	0.93	1.74	2.50	2.18	1.50	1.00	0.50	2.50	0.50	0.50
Bentonite	1.50	1.50	1.50	1.50	1.50	1.50	1.50	1.50	1.50	1.00
Carbohydrases ^1^	0.005	0.005	0.005	0.005	0.005	0.005	0.005	0.005	0.005	0.005
Phytases ^2^	0.010	0.010	0.01	0.01	0.01	0.01	0.01	0.01	0.01	0.01
K Carbonate			0.846	0.656	0.849	0.654	0.408	0.369	0.388	0.325
Limestone	1.357	1.290	1.274	1.261	1.266	1.264	1.200	1.193	1.219	1.259
Monocalcium P	0.667	0.509	0.616	0.576	0.619	0.563	0.484	0.416	0.490	0.435
Salt	0.244	0.106	0.058	0.105	0.059	0.106	0.197	0.190	0.206	0.189
Na bicarbonate	0.018	0.223	0.513	0.447	0.510	0.444	0.302	0.306	0.295	0.322
TiO_2_			0.500	0.500	0.500	0.500	0.500	0.500	0.500	0.500
Vitamins ^3^	0.070	0.070	0.070	0.070	0.070	0.070	0.070	0.070	0.070	0.070
Trace minerals ^3^	0.100	0.100	0.100	0.100	0.100	0.100	0.100	0.100	0.100	0.100
Choline Cl 70%	0.111	0.062	0.142	0.134	0.143	0.133	0.107	0.122	0.104	0.114
L-lysine HCl 78.4	0.331	0.307	0.859	0.703	0.857	0.702	0.451	0.452	0.426	0.438
DL-methionine	0.289	0.251	0.261	0.237	0.260	0.238	0.191	0.194	0.194	0.200
L-threonine	0.133	0.120	0.398	0.323	0.397	0.323	0.198	0.216	0.192	0.202
L-Arginine FB			0.594	0.441	0.593	0.436	0.188	0.168	0.167	0.159
L-Valine	0.100	0.083	0.376	0.291	0.375	0.293	0.150	0.151	0.150	0.152
L-Isoleucine			0.321	0.229	0.321	0.231	0.083	0.095	0.073	0.080
L-Leucine			0.356	0.191	0.356	0.215		0.086		0.015
L-Phenylalanine			0.155	0.063	0.156	0.065		0.009		
L-tryptophan			0.020		0.020					
L-Cystine			0.135	0.114	0.134	0.114	0.068	0.071	0.072	0.079
L-Proline			0.200		0.200					
L-Alanine			0.200		0.200					
L-Glycine			0.391	0.259	0.392	0.256				
L-Aspartic Acid			0.200		0.200					
L-Glutamic acid			0.300		0.300					
L-Glutamine			0.400		0.400					

^1^ Rovabio^®^ Advance T-Flex (xylanase, β-glucanase, and arabinofuranosidase); ^2^ AXTRA ^®^ PHY Gold 10T (Dupont Animal Nutrition) provided 500 FTU/kg; ^3^ Vitamin–mineral concentrate supplied per kilogram of diet: 5040 mg retinol, 17.5 mg cholecalciferol, 105 mg tocopheryl acetate, 4 mg menadione, 4 mg thiamine, 11 mg riboflavin, 77 mg niacin, 18 mg pantothenate, 7 mg pyridoxine, 0.35 mg biotin, 3.0 mg folate, 0.02 mg cyanocobalamin, 23 mg copper, 1.79 mg iodine, 57 mg iron, 171 mg manganese, 0.43 mg selenium, and 143 mg zinc.

**Table 5:** The effects of CP, NE, and E:T on live performance and nutrient utilization ^1^.

T	T Code	Live Performance and Energy Utilization from d19–28									
		WG, g/b/d	FI, g/b/d	FCR	AMEi, kJ/b/d	NEi, kJ/b/d	AMEi/WG, kJ/g	NEi/WG, kJ/g	WG/CP Intake, g/g/b/d	Fat Pad, % d28	N dc d28
1	RP-LNE-LE:T	83.61 ^b^	154.97 ^ab^	1.852 ^ab^	1901 ^b^	1400 ^b^	22.72 ^b^	16.74 ^b^	3.466 ^e^	1.330 ^ab^	0.879 ^a^
2	RP-LNE-HE:T	63.09 ^b^	132.73 ^c^	2.173 ^a^	1664 ^c^	1255 ^c^	27.24 ^a^	20.55 ^a^	2.997 ^f^	1.315 ^ab^	0.860 ^ab^
3	RP-HNE-LE:T	83.08 ^b^	146.33 ^bc^	1.760 ^b^	1891 ^b^	1405 ^b^	22.74 ^b^	16.90 ^b^	3.655 ^cd^	1.223 ^ab^	0.878 ^a^
4	RP-HNE-HE:T	100.17 ^a^	161.45 ^a^	1.614 ^c^	2084 ^a^	1607 ^a^	20.84 ^c^	16.06 ^b^	4.115 ^a^	1.387 ^a^	0.841 ^cd^
5	NP-LNE-LE:T	102.02 ^a^	162.34 ^a^	1.591 ^cd^	1992 ^ab^	1494 ^b^	19.53 ^d^	14.64 ^c^	3.600 ^d^	1.084 ^ab^	0.835 ^cd^
6	NP-LNE-HE:T	100.12 ^a^	160.52 ^a^	1.600 ^c^	1940 ^b^	1467 ^b^	19.34 ^d^	14.62 ^c^	3.752 ^b^	1.074 ^b^	0.832 ^d^
7	NP-HNE-LE:T	101.19 ^a^	155.98 ^ab^	1.542 ^de^	1993 ^ab^	1484 ^b^	19.70 ^d^	14.67 ^c^	3.797 ^b^	1.140 ^ab^	0.823 ^d^
8	NP-HNE-HE:T	103.23 ^a^	157.87 ^a^	1.529 ^e^	2001 ^ab^	1499 ^b^	19.39 ^d^	14.52 ^c^	3.694 ^bc^	1.122 ^ab^	0.853 ^bc^
Pooled SEM		1.79	1.43	0.027	18	14	0.34	0.25	0.040	0.027	0.003
*p*-value											
Treatment		<0.001	<0.001	<0.001	<0.001	<0.001	<0.001	<0.001	<0.001	<0.001	<0.001
Sex covariate		<0.001	0.0089	ns	0.0059	0.0039	ns	ns	ns	ns	0.035

Abbreviations: T, dietary treatment; SEM, standard error of means; RP, reduced crude protein (16% CP); LNE, low net energy (9.9 MJ/kg); HNE, high NE (10.4 MJ/kg); LE:T, low essential amino acid-to-true protein ratio (0.56); HE:T, high E:T ratio (0.60); NP, normal CP (18%); WG, weight gain; FI, feed intake; FCR, feed conversion ratio corrected for mortality (g/g as is); AMEi, apparent metabolizable energy intake; NEi, NE intake; N dc, apparent ileal nitrogen digestibility coefficient; ns, non-significant (*p* > 0.05). ^1^ Each value represents the least squares (LS) mean of 8 replicates, with 16 birds per replicate. ^a–f^ LS means within a column lacking a common superscript differ significantly (*p* < 0.05).

**Table 6:** Effect of CP, NE, and E:T levels on live performance, energy utilization, and carcass traits ^1^.

Trt	Trt Code	Growth Performance and Energy Utilization from d19 to 35										
		WG, g/b/d	FI, g /b/d	FCR	AMEi, kJ/b/d	NEi, kJ/b/d	AMEi/WG, kJ/g	NEi/WG, kJ/g	WG/CP Intake, g/g/b/d	Mortality, %	Breast Yield, % d35	Fat Pad, % d35
1	RP-LNE-LE:T	93.13 ^b^	172.20 ^ab^	1.857 ^a^	2112 ^bc^	1556 ^cd^	22.77 ^bc^	16.77 ^b^	3.465 ^c^	0.78	6.327 ^d^	1.682 ^a^
2	RP-LNE-HE:T	69.26 ^b^	145.82 ^c^	2.130 ^a^	1827 ^c^	1379 ^d^	26.70 ^a^	20.15 ^a^	3.068 ^d^	0.83	6.239 ^d^	1.518 ^ab^
3	RP-HNE-LE:T	90.04 ^b^	163.56 ^bc^	1.869 ^a^	2114 ^ab^	1570 ^bcd^	24.15 ^ab^	17.94 ^ab^	3.460 ^c^	1.56	6.516 ^cd^	1.679 ^a^
4	RP-HNE-HE:T	106.76 ^a^	176.67 ^ab^	1.662 ^b^	2281 ^a^	1758 ^a^	21.46 ^cd^	16.55 ^b^	4.006 ^a^	0.00	7.608 ^bc^	1.629 ^a^
5	NP-LNE-LE:T	112.08 ^a^	181.28 ^a^	1.621 ^b^	2225 ^ab^	1668 ^ab^	19.89 ^e^	14.92 ^c^	3.527 ^bc^	0.00	8.391 ^a^	1.380 ^bc^
6	NP-LNE-HE:T	111.04 ^a^	181.50 ^a^	1.637 ^b^	2194 ^ab^	1659 ^abc^	19.79 ^e^	14.96 ^c^	3.664 ^bc^	0.00	8.536 ^a^	1.383 ^bc^
7	NP-HNE-LE:T	110.39 ^a^	172.65 ^ab^	1.571 ^b^	2206 ^ab^	1643 ^bc^	20.07 ^de^	14.94 ^c^	3.721 ^bc^	1.61	8.355 ^ab^	1.357 ^bc^
8	NP-HNE-HE:T	113.06 ^a^	175.49 ^ab^	1.557 ^b^	2224 ^ab^	1666 ^ab^	19.74 ^e^	14.78 ^c^	3.649 ^bc^	0.78	8.562 ^a^	1.350 ^c^
Pooled SEM		2.08	1.71	0.026	21	16	0.34	0.25	0.040	0.28	0.135	0.026
*p*-value												
Treatment		<0.001	<0.001	<0.001	<0.001	<0.001	<0.001	<0.001	<0.001	ns	<0.001	<0.001
Sex covariate		0.0151	ns	0.0178	ns	ns	0.0191	0.0199	ns	ns	ns	ns

Abbreviations: Trt, dietary treatment; SEM, standard error of means; RP, reduced crude protein (16% CP); LNE, low net energy (9.9 MJ/kg); HNE, high NE (10.4 MJ/kg); LE:T, low essential amino acid-to-true protein ratio (0.56); HE:T, high E:T ratio (0.60); NP, normal CP (18%); WG, weight gain; FI, feed intake; FCR, feed conversion ratio corrected for mortality (g/g as is); AMEi, apparent metabolizable energy intake; NEi, NE intake; ns, non-significant (*P* > 0.05). ^1^ Each value represents the least squares (LS) mean of 8 replicates, with 16 birds per replicate. ^a–e^ LS means within a column lacking a common superscript differ significantly (*p* < 0.05).

**Table 7:** Correlations between the experimental factors and response variables (d19 to 35).

Parameter ^1^	WG	FI	FCR	AMEi	NEi	Breast	Fat Pad	CP	TP	EAA	AME
FI	0.887										
	***										
FCR	−0.949	−0.730									
	***	***									
AMEi	0.876	0.952	−0.748								
	***	***	***								
NEi	0.864	0.930	−0.738	0.987							
	***	***	***	***							
Breast	0.616	0.478	−0.626	0.437	0.457						
	***	***	***	***	***						
Fat pad	−0.361	−0.152	0.407	−0.088	−0.091	−0.435					
	**		***			***					
CP	0.591	0.405	−0.608	0.327	0.270	0.739	−0.588				
	***	***	***	**	*	***	***				
TP	0.583	0.417	−0.593	0.316	0.257	0.739	−0.594	0.977			
	***	***	***	*	*	***	***	***			
EAA	0.444	0.175	−0.492	0.163	0.144	0.689	−0.626	0.866	0.824		
	***		***			***	***	***	***		
AME	−0.007	−0.128	−0.094	0.178	0.194	−0.112	0.205	−0.221	−0.286	−0.049	
									*		
NE	0.062	−0.058	−0.129	0.216	0.312	0.028	0.144	−0.310	−0.368	−0.071	0.856
					*			*	**		***

Abbreviations: WG (g/b/d), weight gain; FI (g as is/b/d), feed intake; FCR (g:g as is), feed conversion ration; AMEi (kJ/b/d), apparent metabolizable energy intake; NEi (kJ/b/d), net energy intake; Breast yield (%); Fat pad %, relative fat pad weight; CP (%), measured dietary crude protein (N × 6.25); measured dietary EAA (%), essential amino acids; TP (%), dietary true protein (measured N × K_A_, ingredient-specific N-to-protein conversion factor); AME (MJ/kg dry matter, DM), measured dietary AME; NE (MJ/kg DM), measured dietary NE. ^1^ Significant probability values are indicated as follows: * *p* < 0.05, ** *p* < 0.01, and *** *p* < 0.001.

## Data Availability

The raw data supporting the conclusions of this article will be made available by the authors on request.
